# Activated volcanism of Mount Fuji by the 2011 Japanese large earthquakes

**DOI:** 10.1038/s41598-023-37735-4

**Published:** 2023-06-29

**Authors:** K. Z. Nanjo, Y. Yukutake, T. Kumazawa

**Affiliations:** 1 Global Center for Asian and Regional Research, University of Shizuoka, 3-6-1 Takajo, Aoi-Ku, Shizuoka, 420-0839 Japan; 2 Center for Integrated Research and Education of Natural Hazards, Shizuoka University, 836 Oya, Suruga-Ku, Shizuoka, 422-8529 Japan; 3 Institute of Statistical Mathematics, 10-3 Midori-Cho, Tachikawa, Tokyo 190-8562 Japan; 4 Japan Agency for Marine-Earth Science and Technology, Yokohama Institute for Earth Sciences, 3173-25 Showa-Machi, Kanazawa-Ku, Yokohama, Kanagawa 236-0001 Japan; 5 Earthquake Research Institute, The University of Tokyo, 1-1-1 Yayoi, Bunkyo-Ku, Tokyo, 113-0032 Japan

**Keywords:** Environmental sciences, Natural hazards, Solid Earth sciences

## Abstract

The relation between earthquakes and volcanic eruptions, each of which is manifested by large-scale tectonic plate and mantle motions, has been widely discussed. Mount Fuji, in Japan, last erupted in 1707, paired with a magnitude (*M*)-9-class earthquake 49 days prior. Motivated by this pairing, previous studies investigated its effect on Mount Fuji after both the 2011 *M*9 Tohoku megaquake and a triggered *M*5.9 Shizuoka earthquake 4 days later at the foot of the volcano, but reported no potential to erupt. More than 300 years have already passed since the 1707 eruption, and even though consequences to society caused by the next eruption are already being considered, the implications for future volcanism remain uncertain. This study shows how volcanic low-frequency earthquakes (LFEs) in the deep part of the volcano revealed unrecognized activation after the Shizuoka earthquake. Our analyses also show that despite an increase in the rate of occurrence of LFEs, these did not return to pre-earthquake levels, indicating a change in the magma system. Our results demonstrate that the volcanism of Mount Fuji was reactivated by the Shizuoka earthquake, implying that this volcano is sufficiently sensitive to external events that are considered to be enough to trigger eruptions.

## Introduction

When earthquakes accompany volcanic eruptions, there is the possibility of a causal relationship between them^1,2^. Many researchers studied how volcanic eruptions are triggered by earthquakes^3–8^, motivated by a physical viewpoint in which the stress^7^ and/or strain^8^ of an eruption needs to change, with earthquakes causing rapid and sometimes large changes in that stress and/or strain. We draw on a specific case in California, where the 1992 Landers earthquake triggered seismicity at very large distances, including the magmatically active Long Valley caldera region, which also experienced a significant coincident deformation transient^5,6^. Another case^1^ complements the search for direct evidence of the pairing of earthquakes and eruptions, having examined the historical record of large earthquakes and explosive eruptions to show that eruptions occurred in the vicinity of a large earthquake more often than is expected by chance^7^. In contrast, many large earthquakes have no immediate effect on volcanoes.

**Figure 1:**
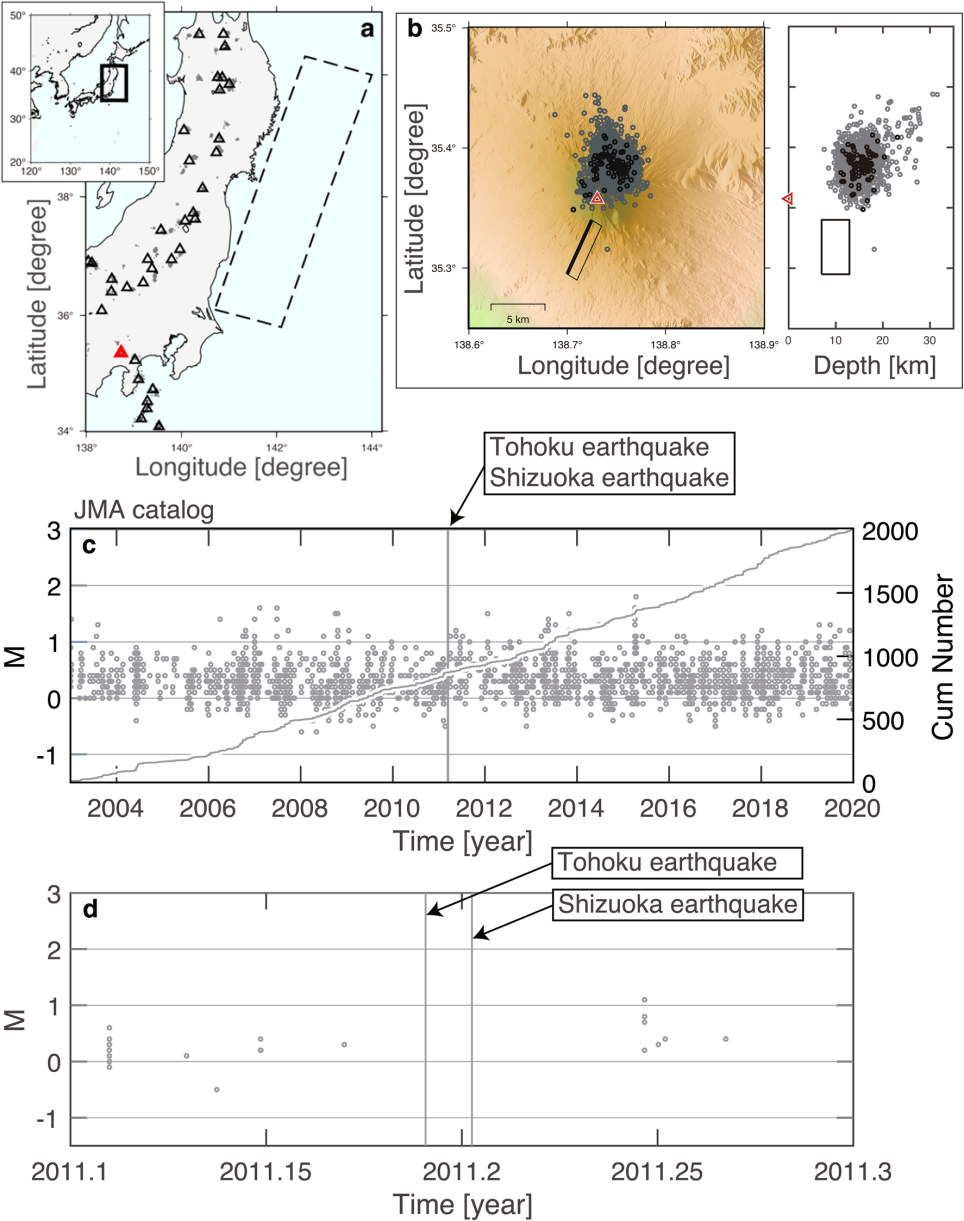
Mount Fuji and Japanese earthquakes. (**a**) Map showing Mount Fuji (red triangle) and the source area of the Tohoku earthquake (rectangular area surrounded by broken lines)^70^. Active volcanoes are indicated by black triangles. Grey dots indicate LFEs in the JMA catalog. (**b**) Left panel shows LFEs (grey circles) around Mount Fuji (summit is indicated by a triangle). Black circles indicate 87 template LFEs. The source area of the Shizuoka earthquake is indicated by a rectangular area^19^. Right panel shows a cross-sectional view of the LFEs and the source area. (**c**) *M*-time diagram of LFEs (y-axis on the left side). Overlapped is the cumulative number of LFEs as a function of time since 2003 (y-axis on the right side). Vertical line indicates the moments of the Tohoku and Shizuoka earthquakes, which overlap. (**d**) Same as (**c**) for a zoom-in plot at times before and after both earthquakes from 2011.1, as a decimal year (Feb. 6, 2011, 12:00:00) to 2011.3, as a decimal year (Apr. 20, 2011, 12:00:00).

The March 11, 2011 magnitude (*M*) 9.0 Tohoku, Japan earthquake and the following large earthquakes that occurred in eastern Japan caused large changes in stress/strain (Fig. 1a). The Japan Meteorological Agency (JMA)^9^ reported active seismicity in about 20 volcanoes after this earthquake, although no eruptions occurred. Recent studies^10–12^ that examined seismic waveforms revealed the detailed behavior of seismic activation beneath the Zao volcano and quiescence beneath the Azuma and Nasudake volcanoes in northeastern Japan after the Tohoku earthquake. Another study^13^, which also examined seismic waveforms, showed remotely-triggered and unrecognized seismicity in the Hakone volcano in central Japan at an epicentral distance of 450 km from the Tohoku earthquake.

Mount Fuji is the Japan’s highest mountain and is also an active volcano. In addition, it has repeatedly erupted, causing lava flows into its vicinity several times. In 1707, the large Hoei eruption during the Hoei era also deposited volcanic ash in Tokyo on the east side of Mount Fuji^14–16^. In the approximately 300 years since that eruption, Mount Fuji has not erupted and volcanic activity has been low, so the risks of eruption-related disasters caused by Mount Fuji have been neglected. However, a swarm of volcanic low-frequency earthquakes (LFEs) from the fall of 2000 to the spring of 2001^17^ changed this situation completely, and the possibility of eruption-related disasters by Mount Fuji has been reassumed while disaster response plans have been developed to counter them^18^. Therefore, it can be stated that the importance of monitoring the volcanic activity of Mount Fuji is increasing.

Mount Fuji exhibited increased seismicity, culminating in the triggered *M*5.9 earthquake that occurred in Shizuoka prefecture 4 days after the Tohoku earthquake. This volcano experienced the 1707 *M*9-class Hoei earthquake 49 days prior to its eruption. This unique experience led to an evaluation of the Tohoku and Shizuoka earthquakes^19^, revealing that crustal deformation due to these earthquakes induced changes in stress of Mount Fuji’s magma reservoir in the order of 0.001–1 MPa. The values are considered to be sufficient to trigger an eruption, if that magma is ready to erupt^20,21^. However, no LFEs were reportedly activated, suggesting that Mount Fuji did not have the potential to erupt^9,19^. It is not easy to characterize LFEs that are ubiquitous and indicative of seismic activities in the deep parts of volcanoes due to their low signal-to-noise ratio^22^.

This study was motivated by previous studies^10–13^, which suggested that the source mechanism of LFEs was quite important to interpret the impact of large earthquakes on the activity of LFEs and deep magmatic processes. We examined if no LFEs beneath Mount Fuji were indeed activated immediately following the Tohoku and Shizuoka earthquakes. A seismic catalog produced in this study revealed that there were LFEs just after the Shizuoka earthquake. Moreover, seismic activation due to this earthquake continued for at least the next eight years, confirmed by using data until 2019. Evidence was shown that the Shizuoka earthquake and crustal deformation^19^ triggered reactivation of the volcano. Given that Mount Fuji is sensitive to such external influences, it is important to carefully monitor its development, especially when events external to the volcano might cause a disturbance.

## Results

In this section, we examine the temporal change in the activity of LFEs beneath Mount Fuji, Japan, and discuss the possibility of an increase in magmatic activities in the lower crust which was triggered by the 2011 Tohoku and Shizuoka earthquakes. We first obtain new catalogs of LFEs, and then apply statistical analyses to examine the temporal changes in LFE activity. Based on the results of these analyses, we conclude that the change in stress caused by the 2011 Shizuoka earthquake enhanced the creation of fractures and resultant change in activity of LFEs around the deep magma reservoir in the lower crust. We also suggest that the activation of LFEs may reflect the sensitivity of Mount Fuji to external disturbances and that the volcano may have the potential to erupt after a 300-year quiescent period.

First, we show the influence of the 2011 Japanese earthquakes on LFEs manifested in the magma system of Mount Fuji. Second, we offer support that this magma system was disturbed due to such events, which played a role on activated LFEs. For inference on volcanic hazards of Mount Fuji, implications for future volcanism based on our observations will be discussed later.

### Influence on LFE activity after the 2011 Japanese earthquakes

To resolve the difficulty in detecting LFEs by conventional event‐detection methods, we produced seismicity catalogs using the matched-filter (MF) method, which cross-correlated a template to continuous seismic signals^22–26^ (details of the “MF method” in “Methods” and Supplementary Figs. S1–S4). In this method, which considers a continuum of seismic signals, an LFE is identified when the timing of a seismic signal and the timing of a template signal overlap. Our resultant catalog includes ~ 16,000 LFEs (Supplementary Fig. S1) whose correlation coefficients (*CC*) to quantify signal similarity range from 0.1 (poor correlation) to 1 (strong correlation and most likely a template LFE). Histograms of *CC*-values show a tall peak at *CC* ~ 0.2 (Supplementary Fig. S1). The minimum threshold for *CC* (*CC*_th_), above which LFEs are used for subsequent analysis, should be above the upper noise limit. A low *CC*_th_ would result in a large number of false detections while a high *CC*_th_ would result in a reduced number of available LFEs. We selected *CC*_th_ = 0.25, at the 2-sigma level of a normal distribution, as is expected for the random correlation between signal and noise^27–29^. Higher values of *CC*_th_ = 0.3, 0.35, and 0.5 above the 3-sigma level were also considered to examine the dependence of the result on *CC*_th_. Mainly the results for *CC*_th_ = 0.25 are shown in the text.

**Figure 2:**
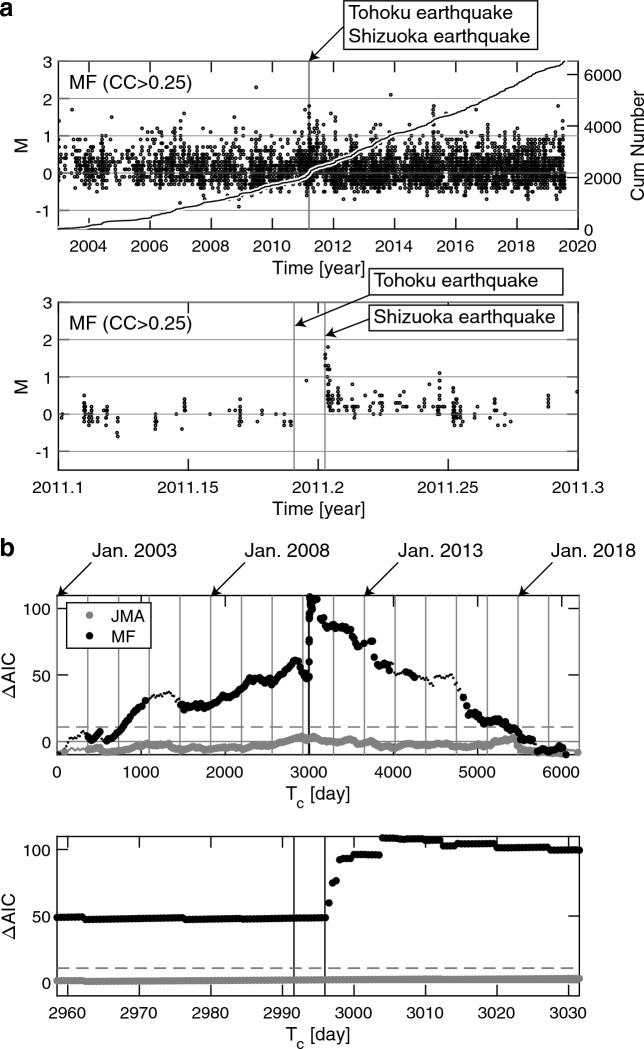
MF method of LFEs. (**a**) Same as Fig. 1c,d for LFEs in the MF catalog (*CC* > 0.25). (**b**) Top panel: ΔAIC = AIC_single_ − AIC_2stage_ as a function of *T*_c_ since 2003, where LFEs (*M* ≥ 0.3) in the MF catalog (*CC* > 0.25) (black) and LFEs (*M* ≥ 0.5) in the JMA catalog (grey) were used. The minimum magnitudes (*M*_th_ = 0.3 and 0.5) for the MF and JMA catalogs, respectively, were used, taking homogeneity of seismicity recordings of both catalogs into consideration (details of catalog quality evaluation in “Methods”). Small points show that the model-fitting analysis did not converge when assuming the corresponding *T*_c_. As a reference, thin vertical lines indicate Jan. 1 for 2004–2019. The timings of the Tohoku and Shizuoka earthquakes overlap, showing a single thick vertical line. Horizontal dashed lines representing 2*q* for the MF and JMA catalogs overlap, where *q* is the degree of freedom imposed when searching *T*_c_ based on the data over the entire period (see “Methods” for details of the ETAS model). Bottom panel: same as the top panel for zoom-in from Feb. 6, 2011, 12:00:00 to Apr. 20, 2011, 12:00:00.

The number of LFEs in our catalog of *CC*_th_ = 0.25 is about three times larger than in the JMA catalog, which lists about 2,000 LFEs detected in 2003–2019 by a conventional method that is not based on *CC* (Figs. 1c and 2a). Our catalogs of *CC*_th_ = 0.25, 0.3, and 0.35 include LFEs immediately after the Shizuoka earthquake (Fig. 2a and Supplementary Fig. S1) while the JMA catalog does not (Fig. 1d). One may see that there are just a handful of LFEs next to the line that indicates the timing of the Shizuoka earthquake (Fig. 2b). There may be doubts as to whether high-frequency (ordinary) aftershocks were mis-detected as LFEs, since the source area of the Shizuoka earthquake and the occurrence area of LFEs were close to each other (Fig. 1b). If *CC*_th_ was selected to be below the upper noise limit, there would be a chance that our catalogs inadvertently included many non-volcanic seismic events. However, our choice of *CC*_th_ (= 0.25, 0.3, 0.35) indicates that events with low-frequency signals truly occurred immediately after the Shizuoka earthquake. Given that the number of LFEs in our *CC*_th_ = 0.5 catalog is about one-fourth of that in the JMA catalog (Supplementary Fig. S1), there were no LFEs immediately after the Shizuoka earthquake.

The cumulative number of LFEs as a function of time in Fig. 2a shows that the rates of occurrence changed around the moments of the Tohoku and Shizuoka earthquakes. To support the observation that either earthquake very likely played a role in reactivation, a statistical analysis was conducted. We introduced the epidemic type aftershock sequence (ETAS) model^30^, assuming that this model, which was originally developed for ordinary earthquakes, was applicable to LFEs (details of the ETAS model in “Methods”). Even though the ETAS model provides a good fit to standard earthquake occurrence, it is known that transient non-standard cases are poorly fitted by the standard ETAS model^31–33^. We focused on differences between the standard ETAS model and an extended two-stage ETAS model, which covers non-standard cases in fitting LFE timeseries, whereas the two-stage ETAS model is the simplest alternative to the standard model^32,33^. This two-stage model assumes different parameter values in subperiods before and after a particular time (change point, *T*_c_), and the whole period is divided into two adjoining periods to fit the ETAS models separately. This is best applied to cases where there is a clear-cut time instant across which ETAS parameter values change^32,33^. To test whether or not there are changes in seismicity pattern at *T*_c_ in a given period, the problem of model selection is reduced by using AIC (Akaike Information Criterion)^34^. We compared AIC_single_ (AIC for a single ETAS fitting) with AIC_2stage_ (AIC for a two-stage ETAS fitting) to select the model with the smaller value, where AIC_2stage_ = AIC_1_ + AIC_2_ (AIC_1_ and AIC_2_ for fitting to the 1st and 2nd subperiods, respectively).

ΔAIC (= AIC_single_ − AIC_2stage_), as a function of *T*_c_ during 2003–2019 (Fig. 2b), shows that the two-stage ETAS model was much better than the single ETAS model, indicating that the most significant *T*_c_ was around the time of the Tohoku and Shizuoka earthquakes. This feature was observed regardless of the *CC*_th_ value (Supplementary Fig. S5). There was a pronounced discontinuation in smoothing at the time of the Shizuoka earthquake due to triggered LFEs for *CC*_th_ = 0.25 (Fig. 2b), 0.3, and 0.35, but this was not the case for *CC*_th_ = 0.5 due to few LFEs around the time of the Shizuoka earthquake. AIC when the JMA catalog was used (grey data in Fig. 2b) also showed a better (but insignificant) outcome when the two-stage ETAS model, rather than the single ETAS model, was used (Supplementary Fig. S5). Namely, ΔAIC was higher than 0 (ΔAIC > 0) when *T*_c_ was around the time of the Shizuoka earthquake, but it was below the horizontal dashed line in Fig. 2b. This line indicates a hurdle to the selection of the two-stage ETAS model, given that *T*_c_ was searched from the data during 2003–2019^32,33^.

**Figure 3:**
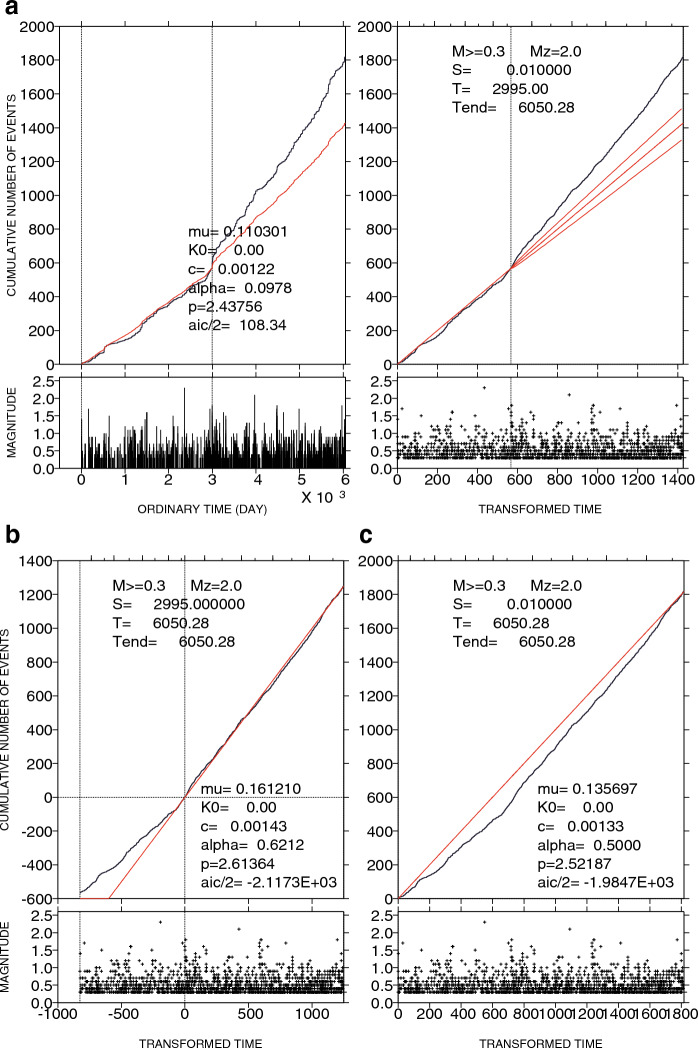
Change point analysis of LFEs. (**a**) Cumulative function of *M* ≥ 0.3 LFEs is plotted against ordinary time (left panel) and transformed time (right panel), showing the ETAS fitting in the target interval from 2003 until immediately before the Shizuoka earthquake and then extrapolated until July 2019. The parabola represents the 95% confidence intervals of the extrapolation. Note that *K*_0_ = 4.51 × 10^–5^ for *M* = 2 (details in the ETAS model in “Methods”), although “*K*_0_ = 0.00” is shown in the graph. The smaller panel below each larger panel indicates an *M*-time diagram. (**b**) As in (**a**) except that the target is the later time interval after the Shizuoka earthquake. Because *K*_0_ = 6.16 × 10^–5^ obtained is too small, it is shown as “*K*_0_ = 0.00” in the graph. (**c**) As in (**a**) except that the target is the entire time interval.

Visual inspection of *T*_c_ immediately before the Shizuoka earthquake confirmed that fitting by the two-stage ETAS model (Fig. 3a,b) was better than that by the single ETAS model (Fig. 3c). An AIC_1_ of 216.7, when seismicity since 2003 until immediately before the Shizuoka earthquake was fitted, shows that the occurrence rates (black) were significantly larger than the extrapolated rates (red) after the Shizuoka earthquake (Fig. 3a). This significant difference continued until more recent times (until 2019). This continuation (AIC_2_ = − 4234.6) (Fig. 3b) was confirmed by switching fitting and extrapolating periods. The fitting of the single ETAS model to seismicity during the entire period (AIC_single_ = − 3969.4) was visually poor (Fig. 3c), with ΔAIC = 48.5 {= − 3969.4–(216.7–4234.6)}.

### Disturbed magma system plays a role on activated LFEs

A challenge in exploring the reason for the changes in the rate of occurrence of LFEs (Figs. 2 and 3) is that the magma system, which is underground, i.e., beneath Mount Fuji, and directly unobservable, results in highly clustered and heterogeneous occurrence times of LFEs. A solution to this problem is to take a reductionism approach, by decomposing LFEs into primary LFEs and secondary-triggered LFEs. Measuring the occurrence rate of the former LFEs (indicative of Poisson background activity) and the latter LFEs (indicative of aftershock activity), allows the magma system and the interactions (triggering) among LFEs to be inferred. The ETAS model can be applied to offer a solution^30–33^. In the ETAS model^30^, seismic activity is expressed by two terms, one for Poisson background activity and another for aftershock activity, assuming that each earthquake (including the aftershocks of another earthquake) is followed by aftershocks (details of the ETAS model in “Methods”).

**Figure 4:**
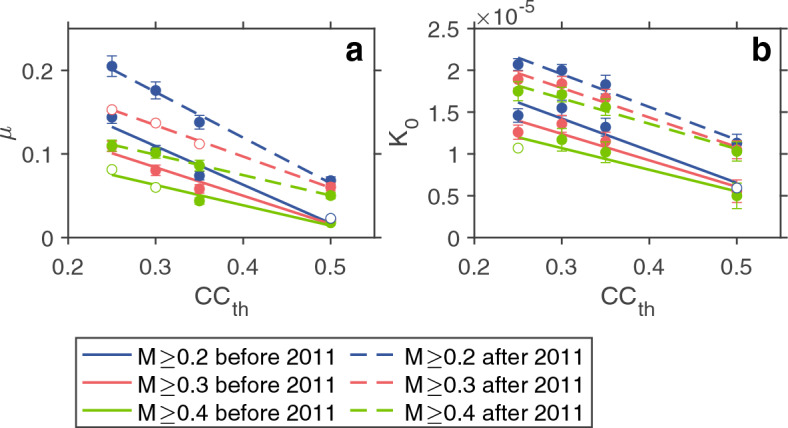
Background and aftershock seismicity. (**a**,**b**) μ and *K*_0_ as a function of *CC*_th_, calculated for two time-windows before Jan. 1, 2011 (solid line) and after Dec. 31 2011 (broken line). We considered the minimum magnitudes *M*_th_ = 0.2 (blue), 0.3 (red), and 0.4 (green). Other ETAS parameters (*c*, *p*, and α) are constants irrespective of different values for time windows, *M*_th_, and *CC*_th_: (*c*, *p*, α) = (0.0015, 2.80, 0.5). Open circles indicate that the model-fitting analysis did not converge well, resulting in large errors. See “Methods” for details of time-dependent μ and *K*_0_ and Supplementary Table S1.

As a preliminary analysis, we examined whether decomposing the LFE sequence was really meaningful, i.e., if secondary triggering played a role in the LFE sequence. Using AIC for the two-stage ETAS model (same model as for Fig. 2b and Supplementary Fig. S5) and the two-stage Poisson model (same as the two-stage ETAS model except that the aftershock activity term was ignored), we compared the goodness-of-fits between them applied to the same dataset. For this comparison, the same procedure as for Fig. 2b and Supplementary Fig. S5 was used except that the single ETAS model was replaced by the two-stage Poisson model. The difference in AIC between the ETAS and Poisson models shows that the former model is superior to the latter one (Supplementary Fig. S6), indicating that the aftershock activity term is not negligible. This result allowed us to examine whether or not the changes in occurrence rate were due to enhanced Poisson background and aftershock activities (Fig. 4 and Supplementary Table S1).

We used μ (a solo parameter of the Poisson background activity term of the ETAS model) and *K*_0_ (a parameter representing clustering aftershock productivity and one of the four parameters of the aftershock activity term), while the other three parameters were constants. For details of these parameters, see the ETAS model in “Methods”. In Fig. 4, μ and *K*_0_ were significantly larger after than before 2011 (details of time-dependent μ and *K*_0_ in “Methods” and Supplementary Table S1). Moreover, μ and *K*_0_ increased with decreasing *CC*_th_ for both periods, before and after 2011. This indicates that small-*CC* LFEs identified by relaxing the event identification protocol contributed to both the Poisson background activity and the aftershock activity. The μ-pattern was not sensitive to switching from *K*_0_ (Fig. 4 and Supplementary Table S1) to other parameters (details of time-dependent μ and *K*_0_ in “Methods” and Supplementary Fig. S7 and Tables S2 and S3). Our results show the contribution of enhanced background and aftershock activities to the increase in occurrence rate of LFEs, and support the possibility of changes in the magma system due to the Shizuoka earthquake.

The bottom panel of Fig. 2a shows that the high rate of LFEs after the Shizuoka event decreased as a function of time, implying that LFEs behaved as aftershocks of the Shizuoka event. Therefore, it may be considered that the background rate of LFEs was globally similar to what was observed before 2011. However, this is not true because μ before 2011 was smaller than after 2011 (Fig. 4a) and because the two-stage ETAS model performed better than the single ETAS model, which included the Shizuoka earthquake (Fig. 2b). A clear change in LFE activity during the occurrence of the Shizuoka earthquake indicates the contribution of the changes in both aftershock and background activities.

**Figure 5:**
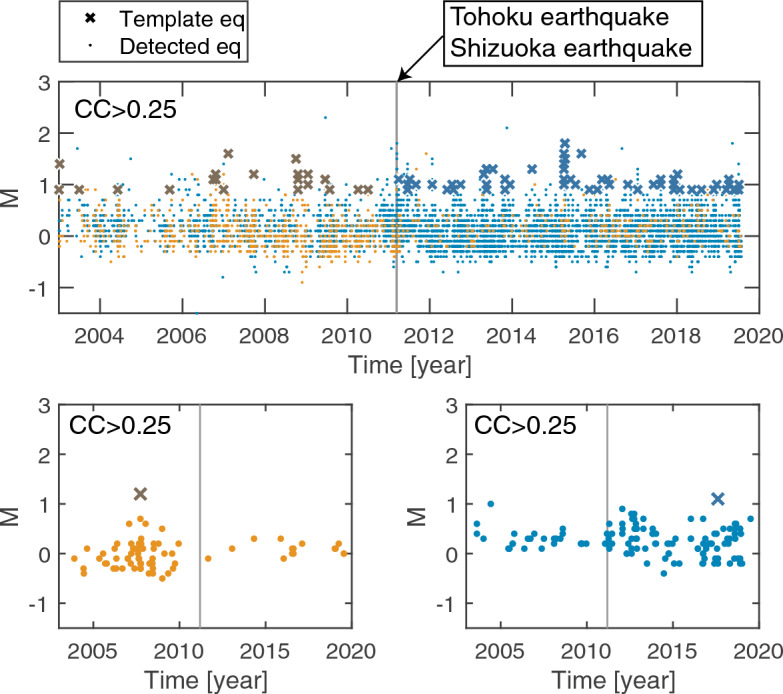
Temporal changes of LFE pattern. Top panel: same as Fig. 2a except for the separation between LFEs (orange dots) detected by a correlation with waveforms of template LFEs (orange cross) listed in the JMA catalog before the Tohoku earthquake and LFEs (blue dots) by a correlation with waveforms of template LFEs (blue cross) after the Shizuoka earthquake. Bottom left panel: same as the top panel for highlighting LFEs detected by using an exemplified template LFE before the Tohoku and Shizuoka earthquakes (vertical line). Bottom right panel: same as the bottom left panel, but after these earthquakes.

If there was to be a disturbance in the magma system, it might induce differences in template LFEs between pre- and post-Shizuoka-quake sequences, allowing differences in detected LFEs between them. A simple test (top panel of Fig. 5) was conducted to assess this possibility, revealing that templates in the pre- (post-) Shizuoka-quake sequence were likely applicable for detecting LFEs in the same sequence. Figure 5 (bottom panels), which shows examples from two individual template LFEs, highlights this difference. The feature was not sensitive to *CC* > 0.25, 0.3, 0.35 and 0.5 (Supplementary Fig. S8). LFEs before/after 2011 were mostly detected by templates in the same time periods.

In this test (top panel of Fig. 5), the two time-windows before and after the Shizuoka earthquake were chosen. It is quite usual for detections to be temporally clustered with template LFEs, so there may be curiosity about the time-windows that were selected. However, the statement that LFEs before/after 2011 were mostly detected by templates in the same time periods is insensitive to selection bias of the time-windows (Supplementary Fig. S9).

## Discussion

A seismic survery^35,36^ that elucidated the 3D structures of the P-wave velocity (*V*_P_), S-wave velocity (*V*_S_) as well as *V*_P_/*V*_S_ beneath Mount Fuji, revealed a low-*V*_P_, low-*V*_S_ and low-*V*_P_/*V*_S_ anomaly at depths of 7–17 km, corresponding with the locations of LFEs. The coincidence of the velocity anomaly and the locations of LFEs suggests that supercritical volatile fluids, such as H_2_O and CO_2_, may be abundant in the low-*V*_P_/*V*_S_ area and may play an important role in generating LFEs.

Two possible processes leading to an eruption are currently considered for Mount Fuji^19^: the promotion of bubbling due to pressurization, and changes in stress in surrounding rocks. The first process promotes exsolving volatile components (H_2_O and CO_2_) from liquid to gas^37–41^. If the magma plumbing system is open or has weak walls that are easily expanded or fractured, increased pressure creates paths for the magma to migrate, so magma pressure will be further reduced and bubbling will be accelerated. In the second process, cracks close to the magma system would be unstable due to stress perturbation. These failures can become paths through which magma flows up, leading to an eruption. Disturbances caused by external fractures, such as the Shizuoka earthquake, may be a key to initiating both processes.

Nakamichi et al.^35^ suggested that a complex process occurred beneath Mount Fuji in which the characteristics of its LFEs were due to a variety of focal processes, and where the source mechanism of the largest LFE (*M*2.3) was explained by non-double-couple components. Among variable LFEs, only those associated with newly-created fractures and reactivated fractures pushed closer to failure by stress changes due to the Shizuoka earthquake, may have become active. The reader might note alternative source processes. LFEs might not always be generated by magma movement. Hydrothermal fluids, as well as the attenuation of higher frequency earthquakes, can create apparent LFEs^42,43^.

**Figure 6:**
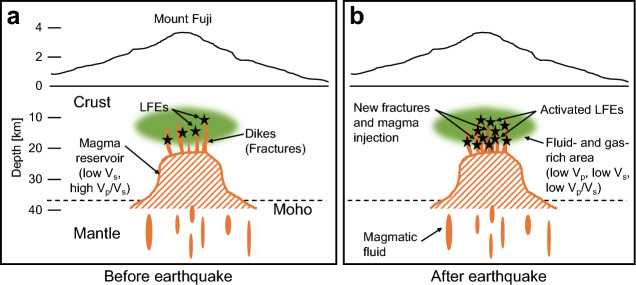
Schematic cross sections. Relationship between LFEs and the magma system beneath Mount Fuji, before and after the Shizuoka earthquake, is shown in (**a**) and (**b**), respectively. (**a**) is based on previous studies^17,35,36,44^. New fractures and magma injection due to the earthquake were suggested^19^, shown in (**b**). We proposed activated LFEs in (**b**).

Schematic cross-sections beneath Mount Fuji^17,44^ before and after the Shizuoka earthquake (Fig. 6) show how newly created fractures^19^ and activated LFEs are associated with changes in the magma system and fluid- and gas-rich area^35,36^. However, based on our result, it remains unsolved if the Shizuoka earthquake triggered an increase in the supply of magmatic fluid to the magma reservoir. Furthermore, there was no evidence that the amount of rising magma increased because clear near-surface deformation around Mount Fuji was not observed before and after the Shizuoka earthquake.

Stress changes on the magma system beneath Mount Fuji of 0.001–0.01 and 0.1–1 MPa were caused by the Tohoku and Shizuoka earthquakes, respectively^19^. The latter change is considered to be sufficient to trigger earthquakes^20,21^, implying the excitement of the magma system and triggering an eruption through either or both processes described above^19^. Our results (Figs. 2, 3, 4, 5) demonstrate that the Shizuoka earthquake played a role in the magma system’s excitation, but not enough to trigger an eruption. We conclude that Mount Fuji was sensitive to disturbances due to this earthquake. This is consistent with a previous study in which it was implied that the crust in the area near Mount Fuji is quite sensitive to transient stress perturbation and that the level of pressurization of the hydrothermal and/or magmatic fluids is high in the Mount Fuji area^45^.

In 1703, four years before the 1707 Hoei eruption, seismic swarms were observed 35 days after the *M*8-class Genroku Kanto earthquake, about 100 km east of Mount Fuji, but no eruption occurred^14–16^. The 1707 eruption was also preceded by the *M*9-class Hoei earthquake, about 200 km to the southwest, on October 7, 49 days before the eruption. Beginning on November 28, 1707, earthquake swarms were observed several times and dozens of earthquakes were felt from December 15, 1707. Mount Fuji then began to erupt on December 16, 1707.

In 2000–2001, LFE swarms occurred, starting in August 2000, two months after the eruption of Miyakejima volcano, which lies 160 km to the south of Mount Fuji, although the change in stress was 10^–4^ MPa^19,46^, or 0.001 ~ 0.0001 of that caused by the Shizuoka earthquake. This change in stress is considered to be too small to trigger an eruption. The experience of Mount Fuji described above implies that it tends to be influenced by external disturbances such as large earthquakes and active volcanoes. Our observation of activated LFEs due to the Shizuoka earthquake and a previous theory of an increase in stress imparted by this earthquake^19^ support this tendency, although the volcano has not yet erupted (June 2023). While this study presents a case for the interaction of the Mount Fuji volcanic system with tectonic earthquakes, it remains possible that considerable volcano-seismic activity took place without any influence by tectonics for some cases.

Over 300 years after the 1707 Hoei eruption, the Japanese government has started to consider preparations for the next eruption, citing a worst-case scenario that inflicts catastrophic damage on humans and society^18^. Whether the increase in occurrence rate of LFEs (Fig. 2) will continue or subside absent external events remains unknown, and it is impossible to draw conclusions about the timing of the next eruption. However, our detailed study demonstrates that LFE activity is an important indicator of Mount Fuji’s subsurface magma system^17,35,36,44^. Given that this study covered data up to 2019, additional analyses for more recent LFEs in future research may be useful for identifying the current state of Mount Fuji. Thus, together with seismological and geodetic observations, it is worthwhile monitoring LFEs to contribute to the prevention and mitigation of Mount Fuji’s volcanic hazard. Our arguments for the use of monitoring LFEs are applicable to active volcanoes around the world that have not yet erupted but are considered to have the potential to erupt.

## Methods

### MF method

When studying LFEs associated with volcanic phenomena, researchers may want to use a catalog that consists of a complete list of LFEs. However, due to their low signal-to-noise ratio, LFE signals are difficult to detect by conventional event-detection methods. We used an MF method that correlates waveforms of continuous signals with those of a template and allows the detection of seismic sequences with a low signal-to-noise ratio^22–26^. In this study, the MF system used for detecting LFEs beneath the Hakone volcano, Japan^22^ was modified so that it was applicable to Mount Fuji.

Waveforms of continuous signals that were used in this study covered the Jan. 2003-Jul. 2019 period, as recorded by 16 seismic stations (Supplementary Fig. S1) with a three-component velocity seismometer around Mount Fuji. These were obtained from the Earthquake Research Institute at the University of Tokyo.

All stations used in this study (Supplementary Fig. S1) host borehole seismometers (eigen frequency of 1 Hz), except for the stations OMZ (35.434332°N, 139.012649°E, 503 m above sea level), FJO (35.3666°N, 138.9102°E, 490 m above sea level), EV.FJZ (35.4487°N, 138.7525°E, 1090 m above sea level), and EV.SBSR (35.36582°N, 138.77818°E, 1980 m above sea level), installed on the surface of the ground. OMZ, FJO, and EV.FJZ host Lennartz seismometers (1 Hz) and EV.SBSR hosts a Nanometrics Trillium seismometer (1/120 Hz).

To prepare template LFEs, we used the JMA catalog, which includes ordinary earthquakes and LFEs observed in Japan. Although ordinary earthquakes are distributed all over Japan, LFEs tend to concentrate beneath active volcanoes (Fig. 1a), along the boundary between the Philippine Sea plate and the continental plate in western Japan^47^, and as several isolated clusters in the intraplate regions^48^. Each event in the JMA catalog is classified based on subsidiary information: natural (ordinary) earthquake, LFE, artificial event, etc. The spatial map of events classified as LFEs shows that the area of LFEs around Mount Fuji was separated from other areas of LFEs (Fig. 1a). We only selected LFEs around Mount Fuji and defined the catalog including these LFEs (Fig. 1b–d). The source area of the Shizuoka earthquake and the occurrence area of LFEs are close to each other (Fig. 1b). However, due to subsidiary information, there is no doubt that the LFE catalog we used eliminated aftershocks (ordinary earthquakes) of the Shizuoka earthquake.

This study relied on statistical analyses of the LFE catalog, which required the use of a complete LFE catalog that covered the study region and time period. It should be carefully considered that the catalog may be controlled by the selection of template earthquakes in the MF analysis. To ensure the completeness of the catalog, it is critical to use a well-chosen set of template LFEs. Careful consideration was needed to make a set of template LFEs, as explained below.

First, large LFEs were selected as templates in order to allow template waveforms to include more information on signals than on noise. Using the JMA catalog, we investigated an *M*-time graph of LFEs around Mount Fuji during the Jan. 2003–Jul. 2019 period (Fig. 1c), and found that the majority was *M* = 0 ~ 1, regardless of the date. Visual inspection shows that LFEs of *M* > 1 were rare with no particular tendency such as LFEs of *M* > 1 which occurred more frequently or less frequently over time. If smaller magnitude criteria were selected to increase the number of template LFEs, then more LFEs would be detected. However, since implementation of the MF system was computationally more intensive when using a large number of template LFEs than when using a small number of template LFEs, so an implementation trial was conducted by using different magnitude criteria under our computing environment. We found that the most feasible was to use *M* ≥ 0.9 LFEs as templates. Thus, irrespective of time, LFEs with *M* ≥ 0.9 in Jan. 2003-Jul. 2019 were selected as templates. Then, among them, LFEs that were recorded by six stations with a minimum signal-to-noise-ratio of 2, were selected^22^.

Second, we verified whether a chosen-set of LFEs (*M* ≥ 0.9) showed appropriate spatiotemporal coverage. This becomes particularly important when the source mechanism changes over time. Map and cross-sectional views around Mount Fuji (Fig. 1b) show that selected LFEs (*M* ≥ 0.9) were mostly distributed within the cluster of LFEs, with all magnitudes between Jan. 2003 and Jul. 2019. A consistent spatial coverage of LFEs (*M* ≥ 0.9) was found between the pre- and post-Shizuoka-quake periods: the coverage of LFEs (*M* ≥ 0.9) before the Shizuoka earthquake was observed in the area where LFEs (*M* ≥ 0.9) had already occurred thus far (Supplementary Fig. S1).

It appears that there were more template LFEs from the post-Shizuoka-quake period than from the pre-Shizuoka-quake period (Fig. 5 and Supplementary Fig. S8), which could potentially result in an overrepresentation of detected earthquakes in the post-Shizuoka-quake period relative to the pre-Shizuoka-quake period. However, as described above, this bias was not intentionally included. Thus, we did not distort to select template LFEs for making a template LFE catalog, which would be used for a subsequent MF procedure.

The MF procedure to identify LFEs, briefly described in this paragraph, is the same as that of Yukutake et al.^22^. Three-component waveform records for each template LFE were used, applying a six-second time window beginning two seconds before the onset time of the theoretical S-wave arrivals. Both templates and continuous waveforms were bandpass-filtered for 1–6 Hz and decimated at 20 Hz to reduce the calculation cost. This band was selected because Yukutake et al.^22^ was followed, although other studies used a slightly narrower band (e.g., 1–4 Hz^49^). The *CC* between a template and continuous waveform at each sampling time for every component at each station was calculated. After subtracting the hypocenter-to-station travel time of the theoretical S-wave, the time sequences of the correlation function throughout all channels were stacked (Supplementary Figs. S2 and S3). When the peak of the stacked correlation function exceeded a threshold level of nine times the median absolute deviation, an event was identified as a candidate LFE. It would be beneficial to show examples of detected LFE waveforms (Supplementary Figs. S2 and S3) in order to verify the earthquake detection process that could enhance the reliability of this study. After removing multiple counts, the location of the candidate was assigned to the hypocenter of the matched template LFE determined by JMA (also see the “Catalog quality evaluation” section in “Methods”). Magnitude was determined as the mean of the maximum amplitude ratios of the template with respect to the candidate. The MF procedure described above was applied to all waveform records in the Jan. 2003–Jul. 2019 period, and a preliminary catalog, including candidate LFEs, was created, but LFEs identified by five or less stations were not included in this catalog^22^.

Less reliable LFEs were removed from this preliminary catalog to create a finalized catalog, as follows. Among candidate LFEs, false detection occasionally occurred due to contamination by other seismic signals such as teleseismic earthquakes. This contamination led to the detection of LFEs with a large *M*, so we visually inspected whether each template LFE was used to detect many candidate LFEs with *M* > 2, a magnitude above which few LFEs have been recorded beneath Mount Fuji in the JMA catalog since 2003. We considered that such template LFEs had a feature similar to teleseismic earthquakes and decided to eliminate them from the list of template LFEs. Thus, candidate LFEs detected by using the eliminated template LFEs were removed from the preliminary catalog, resulting in the finalized catalog that included ~ 16,000 LFEs. A total of 87 template LFEs were used for the finalized catalog. The locations of template LFEs and seismic stations are indicated in Fig. 1b and Supplementary Fig. S1. Despite this primary quality test, an additional test was conducted, as described in the next paragraphs and in the “Catalog quality evaluation” section.

The *CC*-values of LFEs (Supplementary Fig. S1) ranged between 0.1 (poor correlation with a template LFE) and 1 (strong correlation with, and identical to, the corresponding template LFE). Setting the minimum *CC* to a low value implies the use of an incomplete catalog influenced by the nature of low signal-to-noise ratios of LFEs. The minimum threshold for *CC* (*CC*_th_), above which LFEs are used for our analysis, should be above the upper noise limit. We decided to use *CC*_th_ = 0.25, 0.3, 0.35, and 0.5 for the following reasons. Histograms of *CC*-values in Supplementary Fig. S1 show an asymmetric distribution with a tall peak at *CC* ~ 0.2. We followed previous studies^27–29^, in which the distribution of lower *CC*-values was modeled by a normally distributed curve that would be expected for random correlations between signals and noise, while the upper tail was considered to represent the presence of well-correlated LFEs. Visual inspection shows that frequencies at and below *CC* ~ 0.2 are in good agreement with the left-hand side of the normally distributed curve where the mean is 0.19 and its standard deviation is 0.03 (Supplementary Fig. S1). We selected *CC*_th_ = 0.25, which corresponds to the mean plus two standard deviations. Moreover, we selected *CC*_th_ = 0.3 and 0.35, which are higher than the mean plus three standard deviations, to examine the dependence of the result on *CC*_th_. Similar to Green and Neuberg^27^ and Petersen^28^, we found an outliner peak at *CC* ~ 0.3 (Supplementary Fig. S1). This peak was clearly observed in the histogram of *CC*-values for *M* ≥ 0 (Supplementary Fig. S1). This histogram is displayed because our analysis basically did not include LFEs with *M* < 0. Previous researchers, who studied Shishaldin volcanoes (Alaska), the Soufriere volcano (West Indies), and the Unzen volcano (Japan)^27–29^, selected *CC*_th_-values > 0.5, by showing normally distributed curves with larger means and standard deviations than those shown in this study. We also examined the case for *CC*_th_ = 0.5.

The scope of this study did not permit us to reveal repeating LFEs, nor cyclic activities and cluster characteristic, as were studied by Lamb et al.^29^. Rather, this study’s objective was to resolve the difficulty in detecting smaller LFEs. Our future research will be to conduct in-depth analyses of repeating LFEs for each cluster beneath Mount Fuji, referring to Lamb et al.^29^, and using a sophisticated MF method that can locate detected LFEs to appreciate if they occurred in the same cluster as the template LFE used to find them.

### Catalog quality evaluation

As a basis of catalog completeness, understanding magnitude scales used in this study is critical. We examined whether large LFEs in our catalog were indeed large, as in the JMA catalog, and vice versa. An LFE in our catalog was paired with that in the JMA catalog if the time difference between them was within two seconds, while ignoring one-to-multiple cases. In this pairing, differences in the locations of LFEs were not considered because, as described in the “MF method” section in “Methods”, the locations of LFEs in our catalog were assigned to the hypocenter of the matched template LFE determined by JMA. A list of paired LFEs shows that magnitude in our catalog was positively correlated with that in the JMA catalog, and that the former was nearly equal to the latter (Supplementary Fig. S4), allowing us to assume a one-to-one transformation in magnitude between our catalog and the JMA catalog.

Analyses of the ETAS model (see the “ETAS model” section in “Methods”) are critically dependent on a robust estimate of completeness magnitude (*M*_c_) of the processed LFE data. Above *M*_c_, all events are considered to be detected. In particular, underestimates of *M*_c_ lead to unreliable ETAS fitting. Attention always needs to be paid to *M*_c_ when assessing *M*_c_ in each time window. Details of how to compute *M*_c_ are provided in the “Computation of *M*_c_” section in “Methods”.

*M*_c_ was about 0.3 ~ 0.5 for the JMA catalog and about 0.2 ~ 0.3 for the MF catalogs (Supplementary Fig. S4). These estimates of *M*_c_ were based on precut catalogs covering several time periods (Supplementary Fig. S4). Therefore, we did not consider a single *M*_c_ over the entire catalog. To verify whether the results depended on the choice of minimum magnitude (*M*_th_), above which the ETAS model was fitted, we assumed *M*_th_ = 0.2, 0.3, and 0.4 (*M* ≥ 0.2, 0.3 and 0.4), suggesting that the feature generally appears to remain stable (see the “ETAS model” section in “Methods”, Figs. 2, 3 and 4, and Supplementary Figs. S5–S7 and Tables S2 and S3). Visual inspection of Fig. 2 shows that the catalog for *M* = 0 or less is affected by incompleteness. However, it was not necessary to account for such small LFEs because *M*_th_ = 0.2, 0.3, and 0.4 were assumed for the ETAS analyses. For the JMA catalog, *M*_th_ = 0.3, 0.4, and 0.5 were used (Fig. 2 and Supplementary Fig. S5).

### Computation of *M*_c_

To compute *M*_c_, we used the Gutenberg-Richter (GR) relation^50^, given by log_10_*N* = *a*-*bM*, where *N* is the cumulative number of earthquakes with a magnitude larger than or equal to *M*, *a* characterizes seismic activity or earthquake productivity of a region, and constant *b* is used to describe the relative occurrence of large and small events (i.e., a high *b*-value indicates a larger proportion of small earthquakes, and vice versa). Changes in *b*-values of ordinary earthquakes are known to reflect structural heterogeneity, strength, and temperature in the Earth^51–55^, and the *b*-value is also known to be inversely dependent on differential stress^56,57^. We assumed that the GR relation was applicable to not only ordinary earthquakes^55,58,59^ but also LFEs. In this section, the word “earthquake” includes LFEs.

We employed the Entire-Magnitude-Range (EMR) technique^60^, which simultaneously calculates the *a*- and *b*-values and the completeness magnitude *M*_c_, above which all events are considered to be detected. We always paid attention to a robust estimate of *M*_c_, because it is critical for analyses of the ETAS models (details of catalog quality evaluation in “Methods”). EMR applies the maximum-likelihood method when computing the *b*-value to events with magnitudes above *M*_c_. Uncertainty in *b* was according to Shi and Bolt^61^. Substitution of *M*_c_, *N* at *M*_c_, and the maximum-likelihood *b*-value into *M*, *N*, and *b* of *a* = log_10_*N* + *bM*, respectively, gives the *a*-value. Supplementary Fig. S4 shows a good fit of the GR relation to observations in the present cases.

To compute *M*_c_, the EMR technique^60^ combines the GR relation with a detection rate function. Details are provided next. Statistical modeling was performed separately for completely detected and incompletely detected parts of the frequency-magnitude distribution. The *b*- and *a*-values in the GR relation were computed based on earthquakes above a certain magnitude (*M*_cc_). For earthquakes whose magnitudes were smaller than *M*_cc_, it was hypothesized that the detection rate depended on their magnitudes in such a way that large earthquakes were almost entirely detected while smaller ones were detected at lower rates. Earthquakes with *M* ≥ *M*_cc_ were assumed to be detected with a detection rate of 1. To evaluate the fitness of the model to data, the log-likelihood was computed by changing the value of *M*_cc_. The best fitting model was that which maximized the log-likelihood value.

The software package ZMAP^62^ was used to facilitate the computation of *a*, *b*, and *M*_c_ based on the EMR method. Although the package, whose code is open, is written in Mathworks’ commercial software language Matlab®, no knowledge is needed since ZMAP is GUI-driven. ZMAP combines many standard seismological tools. A user can use ZMAP to create a graph of frequency-magnitude distribution with the GR relation with *a*, *b*, and *M*_c_ values calculated by EMR (Supplementary Fig. S4).

### ETAS model

The ETAS model^30^ was originally introduced for ordinary earthquakes, but we assumed that the model can be extended and applied for LFEs beneath Mount Fuji. In this section, when the word “earthquake” is used, the reader should understand that it also includes LFEs.

The ETAS model is a point-process model that represents the activity of earthquakes of a minimum magnitude (*M*_th_) and above in a certain region during a specified time interval. Seismic activity includes the background activity at a constant occurrence rate μ (Poisson process). The model assumes that each earthquake (including the aftershock of another earthquake) is followed by aftershocks. Aftershock activity is represented by the Omori-Utsu formula^63^ in the time domain. The rate of an aftershock occurrence at time *t* following the *i*-th earthquake (time *t*_*i*_ and magnitude *M*_*i*_) is given by ν_*i*_(*t*) = *K*_0_exp{α(*M*_*i*_-*M*_th_)}(*t*-*t*_*i*_ + *c*)^-*p*^ for *t* > *t*_*i*_, where *K*_0_, α, *c*, and *p* are constants, which are common to each target aftershock sequence in a region. The rate of occurrence of the whole earthquake series at *t* becomes $\lambda \left(t|{H}_{t}\right)=\mu +{\sum }_{S<{t}_{i}<t}{\nu }_{i}\left(t\right)$. The summation is performed for all *i* satisfying *t*_*i*_ < *t*. Here, *H*_*t*_ represents the history of occurrence times with associated magnitudes from the data {(*t*_*i*_, *M*_*i*_)} before time *t*. The parameter set θ = (μ,* K*_0_, α, *c*, *p*) represents the characteristics of seismic activity. The units of the parameters are day^−1^, day^−1^, no unit, day, and no unit, respectively. For the case of *K*_0_ = 0, the ETAS model reduces to the Poisson model (Supplementary Fig. S6). We estimated these parameters using the maximum likelihood method. Because *K*_0_ depends on *M* in this model, it is necessary to assume a magnitude at which a value for *K*_0_ needs to be known. Throughout this study, *M* = 2 was assumed for estimating *K*_0_.

Using the maximum likelihood estimate, it is possible to visualize how well or poorly the model fits an earthquake sequence by comparing the cumulative number of earthquakes with the rate calculated by the model. If the model presents a good approximation of observed seismicity, an overlap with each other is expected. Ordinary time can be converted to transformed time in such a way that the transformed sequence follows the Poisson process (uniformly distributed occurrence times) with unit intensity (occurrence rate) so that visualization can be achieved in two ways^31–33^: one graph using ordinary time and the other using transformed time (Fig. 3). Included in the latter graph is the parabola of 95% significance. When the number of earthquakes is insufficiently large, significance actually depends on sample size due to the estimation accuracy of the parameters. The significance of deviation is defined in the case where the empirical curve deviates outside the parabola.

A FORTRAN program package (SASeis2006) associated with a manual for the ETAS analysis was used to calculate maximum likelihood estimates and also to visualize model performance^64^. This has been extended to the program package XETAS^31^ using GUI.

When the stationary ETAS model does not fit a dataset well, the simplest alternative model is a “two-stage ETAS model” that considers different parameter values in subperiods before and after a particular time, referred to as change-point *T*_c_. AIC is used to test whether or not the changes in seismicity pattern at *T*_c_ reduces model selection^34^. In this procedure, we separately fitted the ETAS models for each divided period and then compared their total goodness-of-fit values against the one-fit value over the whole period using the principle of minimum AIC. AIC was calculated from the maximum log-likelihood and number of adjusted parameters.

If *T*_c_ is hypothetically prefixed based on some information other than the occurrence data themselves, such as a notable geophysical event or a notable outside large earthquake, AIC_single_ (AIC for the model fitted over the whole period) can be compared with AIC_2stage_ (AIC for the 2-stage model fitted on divided periods) to select the model with the smaller value that performs a better fit to the data in the entire target period. If *T*_c_ is searched from the target data, the 2-stage model becomes harder to accept. Namely, AIC_2stage_ is compared with AIC_single_ plus the penalty term 2*q* to select the model. Here *q* is the degree of freedom to search for the best candidate *T*_c_ from the data. *q* depends on sample size (number of earthquakes in the target period)^33,65^: *q* increases with sample size and, for example, lies in the 4–5 range for *q* between 100 and 1000.

Although we adopted AIC for model selection, it cannot always be used for other cases such as the identification of possible earthquake precursors in ionospheric electric content (TEC)^66^.

### Time-dependent *μ *and *K*_0_

The standard stationary ETAS model can be temporally extended to the non-stationary ETAS model^33,67–69^ in such a way that μ and *K*_0_ are assigned as a function of *t*. The function μ(*t*) and *K*_0_(*t*) are represented by a broken line connecting the respective sequences (*t*_*i*_, μ(*t*_*i*_)) and (*t*_*i*_, *K*_0_(*t*_*i*_)) for the *i*-th earthquake, using a Bayesian function.

Although such a sophisticated model is available, a simpler approach was taken to capture essential aspects of the time-dependent background and aftershock activities (Fig. 4 and Supplementary Fig. S7 and Tables S1–S3). This involves taking a time-window approach. In Fig. 4, the time-windows 2003–2010 and 2012–2019 were considered where the time-window of 2011, which included the Tohoku and Shizuoka earthquakes, was not considered. When creating Fig. 4 (Supplementary Table S1), μ and *K*_0_ were computed, given that other parameters (α, *c*, *p*) were constants, where the values for (α, *c*, *p*) were obtained as follows. The parameters θ = (μ, *K*_0_, α, *c*, *p*) were first computed for each time-window, each *M*_th_, and each *CC*_th_. Typical values for α, *c*, *p* were α = 0.5, *c* = 0.0015, and *p* = 2.8. Using these typical values, parameters μ and *K*_0_ were recomputed for each time-window, each *M*_th_, and each *CC*_th_. The error bars for μ and *K*_0_, which can be calculated by XETAS^31^, are based on error distribution depending on the sample size when the number of LFEs is not large enough^65^.

*K*_0_ was forced to express time-dependent aftershock activity (Fig. 4), so the same analysis can be performed for other parameters to support the assumption that the μ-*CC*_th_ pattern generally remains stable. Namely, *p* was considered as a time-variable parameter, given that other aftershock parameters (*K*_0_, α, *c*) were prefixed as constant, resulting in Supplementary Fig. S7. The same analysis was performed by assuming time-variable α, given that *K*_0_, *c*,* p* were constant (Supplementary Fig. S7). The parameter values are summarized in Supplementary Tables S1–S3.

The slope (*g*) and intersection (*h*) of the least-square regression line and the square of the sample correlation coefficient (*R*^2^) for Fig. 4a are μ = *gCC*_th_ + *h* with (*g*, *h*, *R*^2^) = (− 0.46, 0.25, 0.96) for *M* ≥ 0.2 (blue solid line), (− 0.34, 0.19, 0.96) for *M* ≥ 0.3 (red solid line), and (− 0.24, 0.14, 0.95) for *M* ≥ 0.4 (green solid line) before 2011, and (− 0.54, 0.34, 0.99) for *M* ≥ 0.2 (blue dashed line), (− 0.37, 0.25, 1.00) for *M* ≥ 0.3 (red dashed line), and (− 0.24, 0.17, 1.00) for *M* ≥ 0.4 (green dashed line) after 2011.

Similarly, for Fig. 4b, *K*_0_ = *gCC*_th_ + *h* with (*g*, *h*, *R*^2^) = (− 3.84 × 10^–5^, 2.58 × 10^–5^, 0.91) for *M* ≥ 0.2 (blue solid line), (− 3.16 × 10^–5^, 2.19 × 10^–5^, 0.90) for *M* ≥ 0.3 (red solid line), and (− 2.59 × 10^–5^, 1.85 × 10^–5^, 0.87) for *M* ≥ 0.4 (green solid line) before 2011, and (− 3.93 × 10^–5^, 3.13 × 10^–5^, 0.97) for *M* ≥ 0.2 (blue dashed line), (− 3.53 × 10^–5^, 2.85 × 10^–5^, 0.97) for *M* ≥ 0.3 (red dashed line), and (− 3.03 × 10^–5^, 2.57 × 10^–5^, 0.97) for *M* ≥ 0.4 (green dashed line) after 2011.

## Supplementary Information

Supplementary Information.

## Data Availability

The datasets used and/or analyzed during the current study are available from the corresponding author on reasonable request. The JMA catalog was obtained from https://www.data.jma.go.jp/eqev/data/bulletin/hypo.html. The waveform records were obtained from the permanent stations of the National Research Institute for Earth Science and Disaster Resilience, the Earthquake Research Institute at the University of Tokyo, JMA, and the Hot Springs Research Institute of Kanagawa Prefectural Government. Locations of active volcanoes used for Fig. 1a were obtained from https://www.mri-jma.go.jp/Dep/sei/fhirose/plate/en.PlateData.html. The fault model of the 2011 Tohoku earthquake, used to create Fig. 1a, was obtained from Asano et al.^70^. The fault model of the 2011 Shizuoka earthquake, used to create Fig. 1b and Supplementary Fig. S1, was obtained from Fujita et al.^19^. The seismicity analysis software ZMAP^62^, used for Supplementary Fig. S4, was obtained from http://www.seismo.ethz.ch/en/research-and-teaching/products-software/software/ZMAP. The program XETAS^31^, used for Figs. 2–4 and Supplementary Figs. S5–S7 and Tables S2 and S3, was obtained from http://evrrss.eri.u-tokyo.ac.jp/software/xetas/index.html. Generic Mapping Tools (GMT)^71^, used for Fig. 1a,b and Supplementary Fig. S1, is an open-source collection (https://www.generic-mapping-tools.org).
