# Supplementary material for: Activated volcanism of Mount Fuji by the 2011 Japanese large earthquakes
Source: Sci Rep. 2023 Jun 29;13:10562. doi: 10.1038/s41598-023-37735-4 (PMC10310753; doi:10.1038/s41598-023-37735-4)
Supplement: Supplementary file 1 — Supplementary Information. [file 41598_2023_37735_MOESM1_ESM.docx]

Supplementary Materials for

Activated volcanism of Mount Fuji by the 2011 Japanese large earthquakes

K. Z. Nanjo*, Y. Yukutake, T. Kumazawa

*Correspondence to: nanjo@u-shizuoka-ken.ac.jp

**This file includes:** Supplementary Figs. S1-S9 and Tables S1-S3.


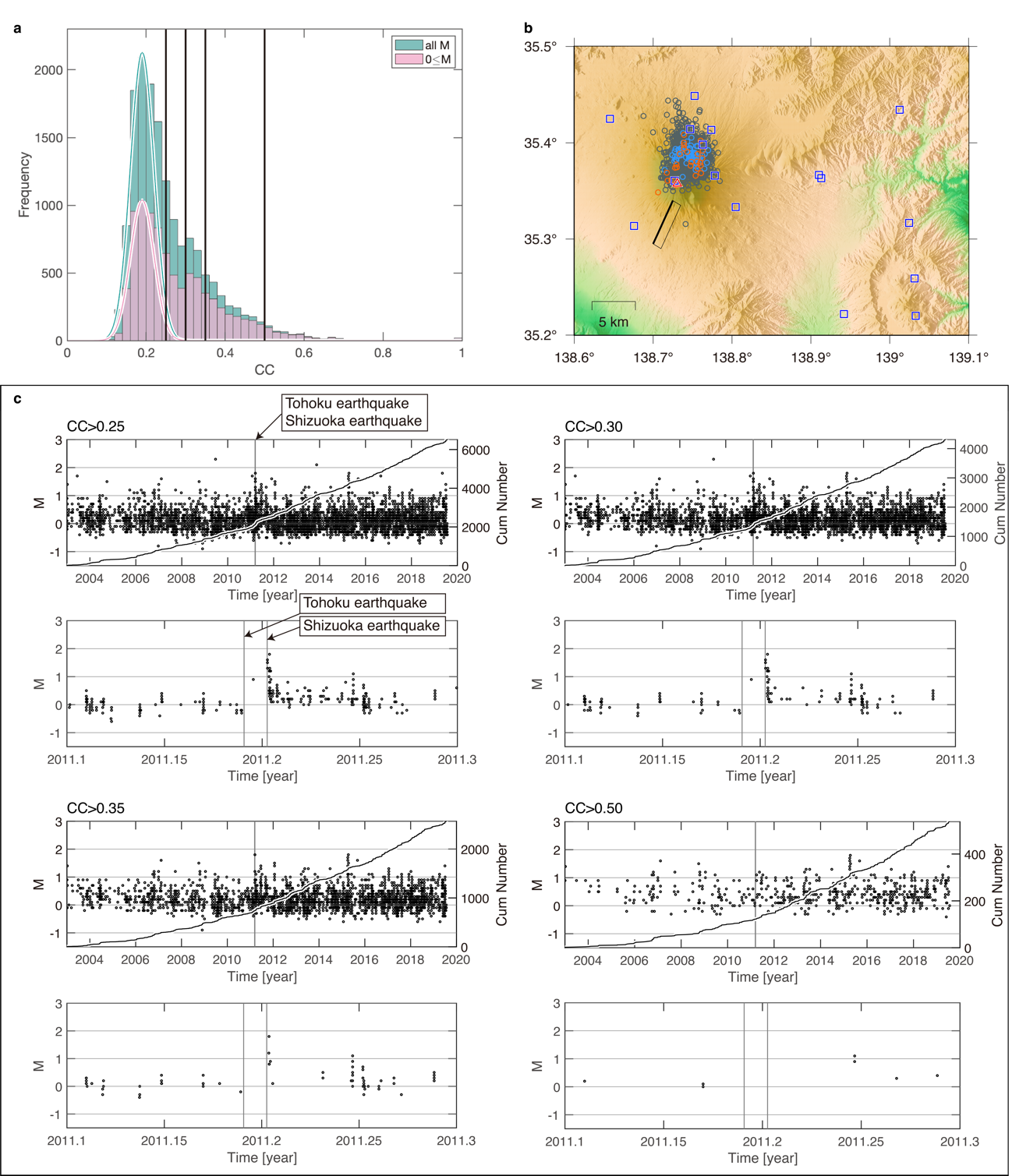


**Supplementary Fig. S1.** Produced LFE catalogs. **a**, Multiple histograms of *CC*-values for all magnitudes (green) and 0≤*M* (pink). Also included are the normally distributed curves (mean of 0.19 and standard deviation of 0.03). Vertical lines indicate *CC*=0.25, 0.3, 0.35, and 0.5. **b**, Spatial map of LFEs. Grey circles indicate all LFEs around Mount Fuji (summit is indicated by a triangle) in the JMA catalog. Template LFEs selected from the JMA catalog before and after the Shizuoka earthquake are indicated by orange and blue circles, respectively. Squares indicate 16 seismic stations. Rectangular area indicates the source area of the Shizuoka earthquake^19^. **c**, *M*-time diagrams. Top left panels: same as Fig. 2**a**,**b** (*CC*>0.25). In the x-axis of the lower graph, decimal years are used. For example, 2011.1 and 2011.3 represent Feb. 6, 2011, 12:00:00 and Apr. 20, 2011, 12:00:00, respectively. Top right panels, bottom left panels, and bottom right panels: same as the top left panels for *CC*>0.3, 0.35, and 0.5, respectively.


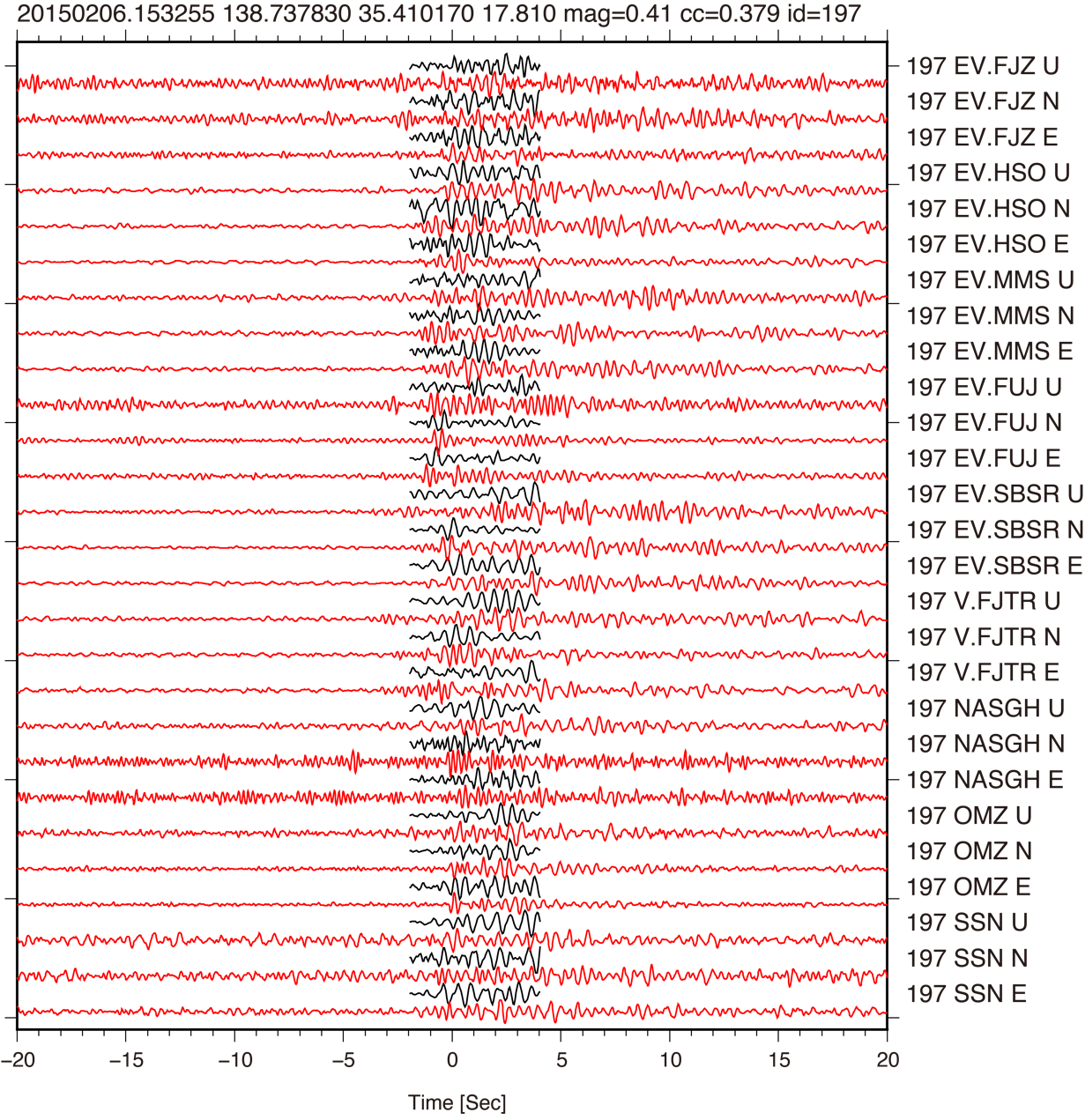


**Supplementary Fig. S2.** Example of the MF method. Continuous waveforms (red) and their matched template waveforms (black) at each channel near the arrival times of the detected event. Event information is as follows. Time: Feb. 6, 2015, 15:32:55; assigned location: 138.73783°E, 35.41017°N, and depth 17.81 km; *M*=0.4; *CC*=0.379; and template event ID 197. Station names with three components (U, N, and E) used to detect the event are given on the right axis.


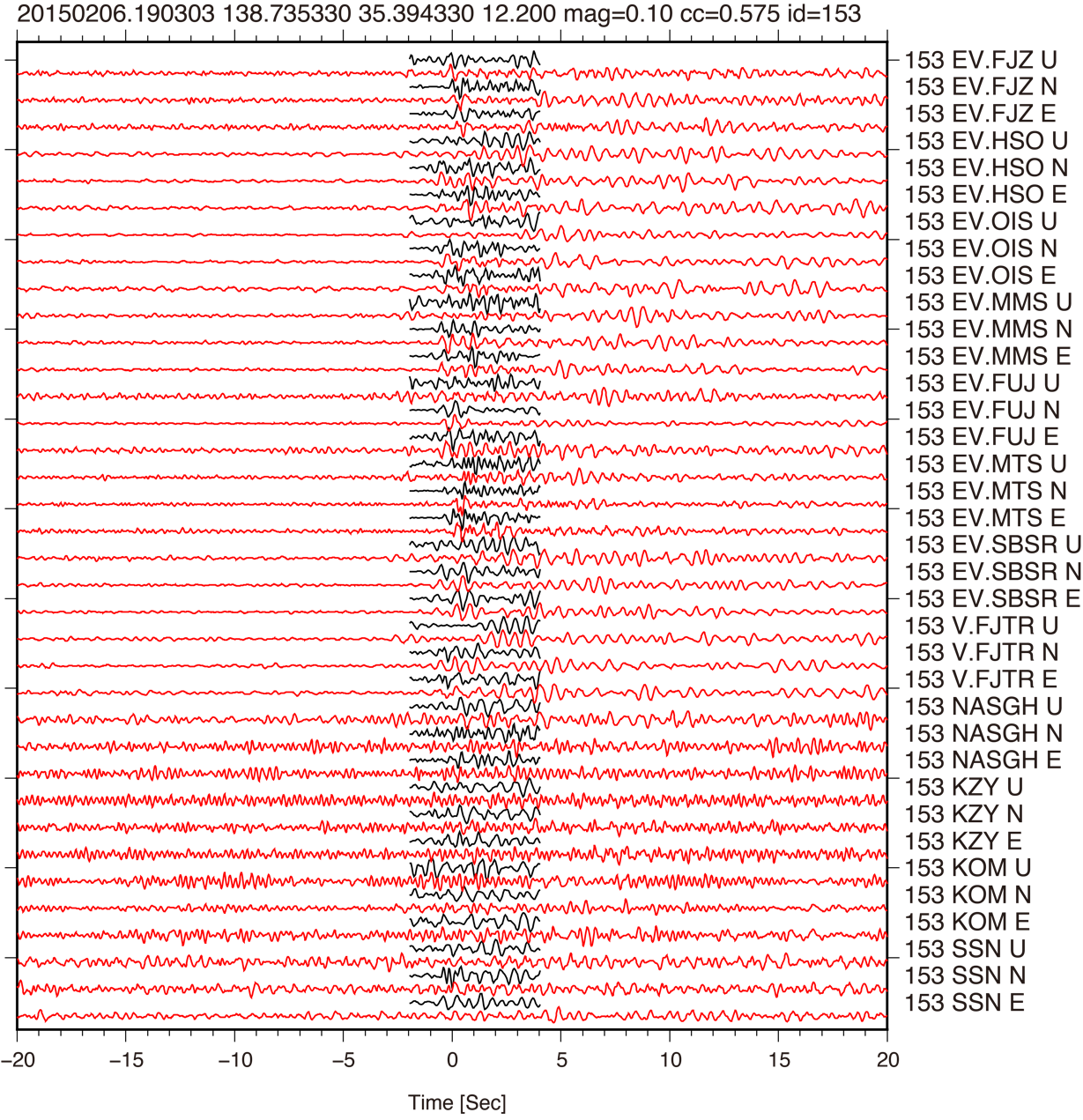


**Supplementary Fig. S3.** Same as Supplementary Fig. S2 for another example.


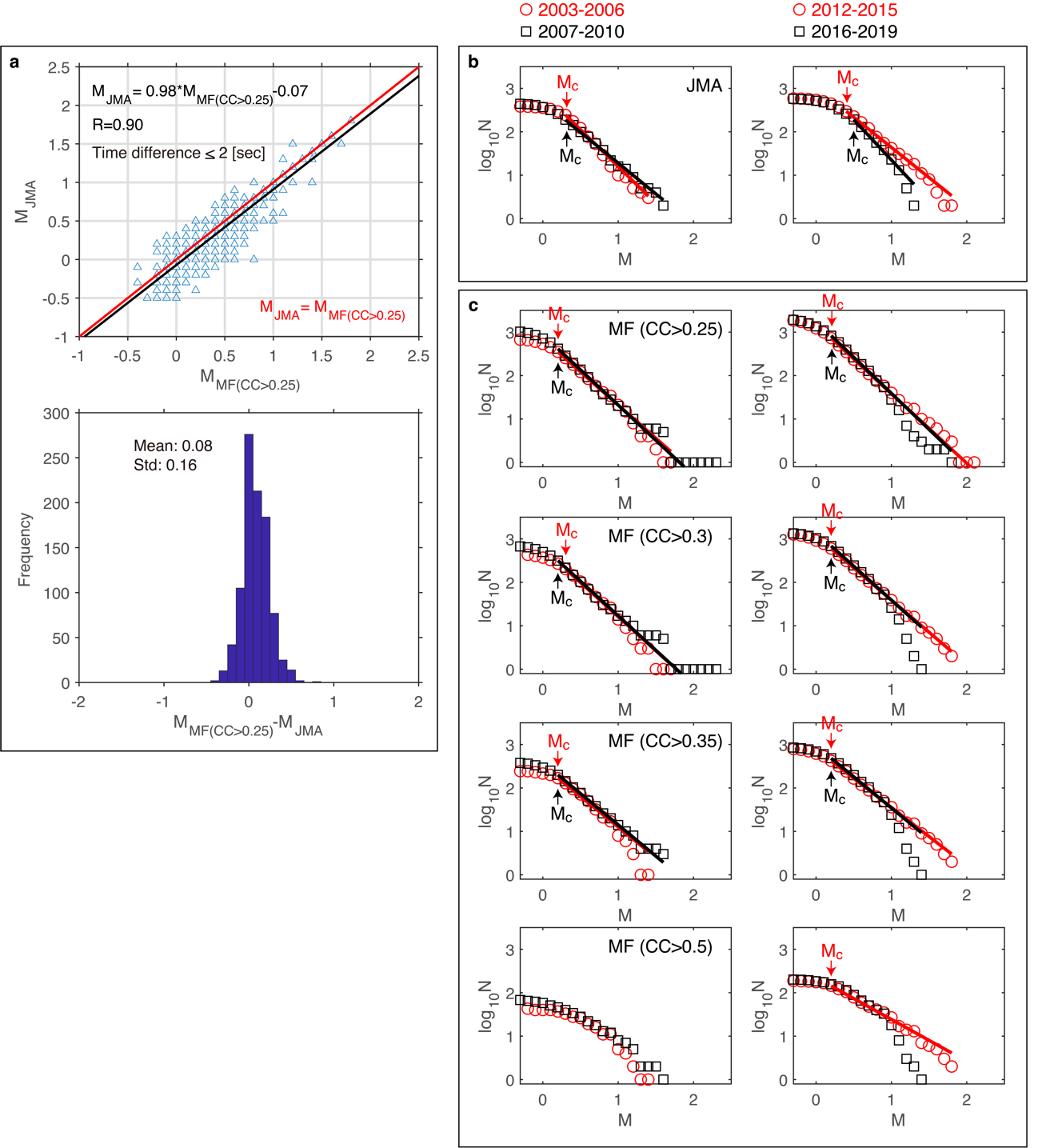


**Supplementary Fig. S4.** Comparison between the JMA and MF catalogs. **a**, Top panel: Magnitude for the JMA catalog, *M*_JMA_ vs. magnitude for the MF catalog (*CC*>0.25), *M*_MF(_*_CC_*_>0.25)_ (details of catalog quality evaluation in Methods). Black and red lines indicate the least-square regression line and *M*_JMA_=*M*_MF(_*_CC_*_>0.25)_, respectively. Bottom panel: Histogram of *M*_MF(_*_CC_*_>0.25)_-*M*_JMA_. Mean and standard deviation of *M*_MF(_*_CC_*_>0.25)_-*M*_JMA_ are 0.08 and 0.16, respectively. **b**, Frequency magnitude distribution of LFEs and *M*_c_ for the several time periods, based on the JMA catalog. (*b*, *a*, *M*_c_)=(1.70±0.09, 2.90, 0.3) for 2003-2006, (1.42±0.09, 2.70, 0.3) for 2007-2010, (1.39±0.07, 3.03, 0.4) for 2012-2015, and (1.86±0.10, 3.21, 0.5) for 2016-2019. **c**, Same as **b** for the MF catalog. Top panels (*CC*>0.25): (*b*, *a*, *M*_c_)=(1.53±0.08, 2.85, 0.2), (1.62±0.08, 2.94, 0.2), (1.60±0.06, 3.18, 0.2), and (1.66±0.05, 3.25, 0.2) for 2003-2006, 2007-2010, 2012-2015, and 2016-2019, respectively. Second panels from the top (*CC*>0.30): (*b*, *a*, *M*_c_)=(1.57±0.10, 2.78, 0.3), (1.59±0.09, 2.82, 0.2), (1.48±0.06, 3.07, 0.2), and (1.56±0.05, 3.15, 0.2), for 2003-2006, 2007-2010, 2012-2015, and 2016-2019, respectively. Second panels from the bottom (*CC*>0.35): (*b*, *a*, *M*_c_)=(1.41±0.00, 2.51, 0.2), (1.44±0.10, 2.59, 0.2), (1.34±0.06, 2.89, 0.2), and (1.43±0.05, 2.97, 0.2), for 2003-2006, 2007-2010, 2012-2015, and 2016-2019, respectively. Bottom panels (*CC*>0.50): (*b*, *a*, *M*_c_)=(0.965±0.07, 2.35, 0.2) for 2012-2015. For 2003-2006, 2007-2010, and 2016-2019, the GR straight line is not shown because the number of LFEs yielding a fit to the GR law is <50.


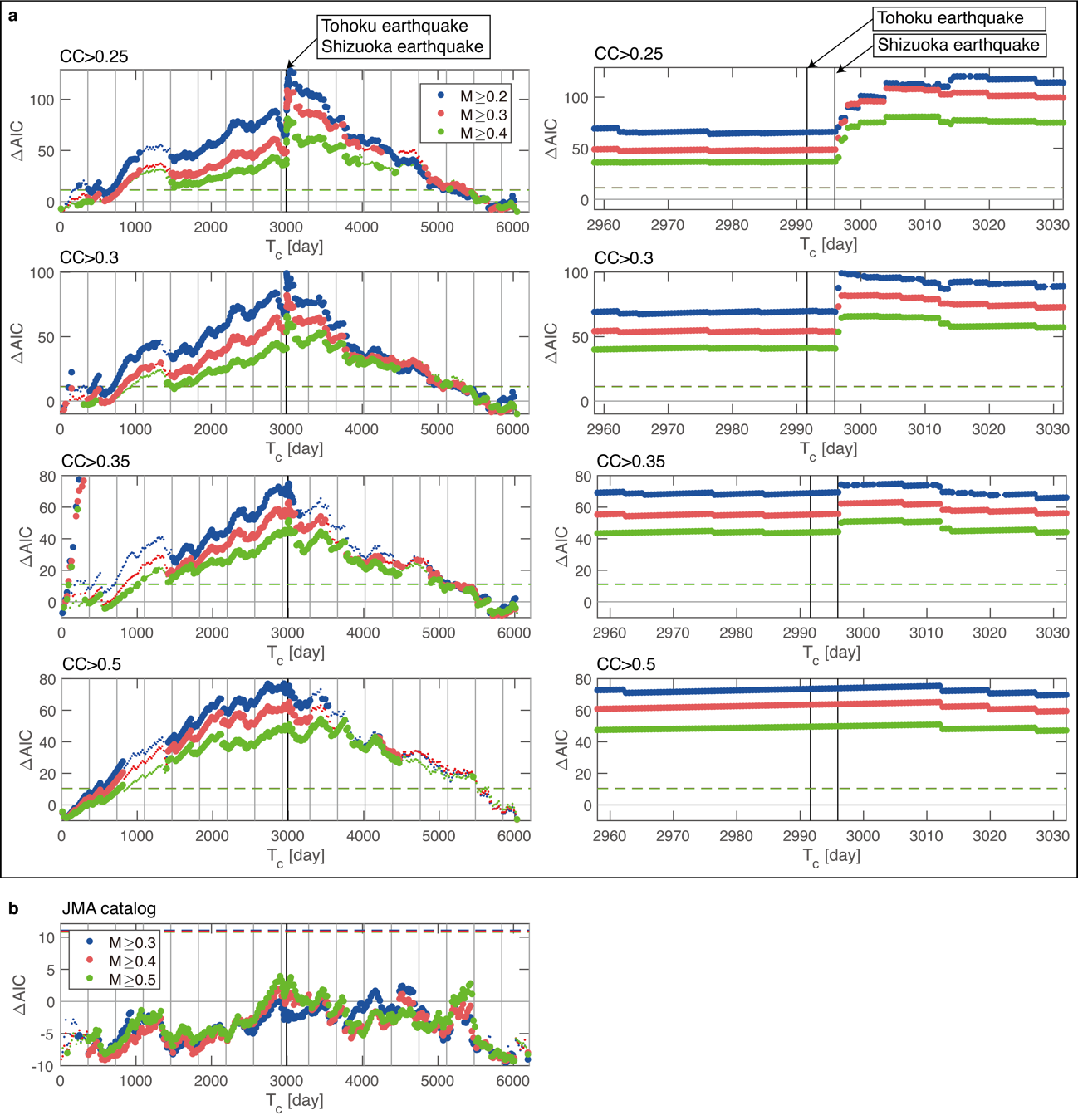


**Supplementary Fig. S5.** Change point analysis. **a**, Left panels: ΔAIC as a function of *T*_c_ for different minimum magnitudes (*M*_th_=0.2, 0.3, and 0.4) of LFEs obtained for the MF method (*CC*>0.25, 0.3, 0.35, and 0.5). Right panels: Same as the left panels for zoom-in at times before and after the Tohoku and Shizuoka earthquakes. In the left panel of *CC*>0.35, ΔAIC-values above the horizontal dashed lines for *T*_c_≤290 were interpreted to be less reliable because the model-fitting analysis converged to a local optimal solution. **b**, Same as the left panels of **a** for the JMA catalog (*M*_th_=0.3, 0.4, and 0.5). For *M*_th_, see the “Catalog quality evaluation” section in Methods. Also see the caption of Fig. 2.


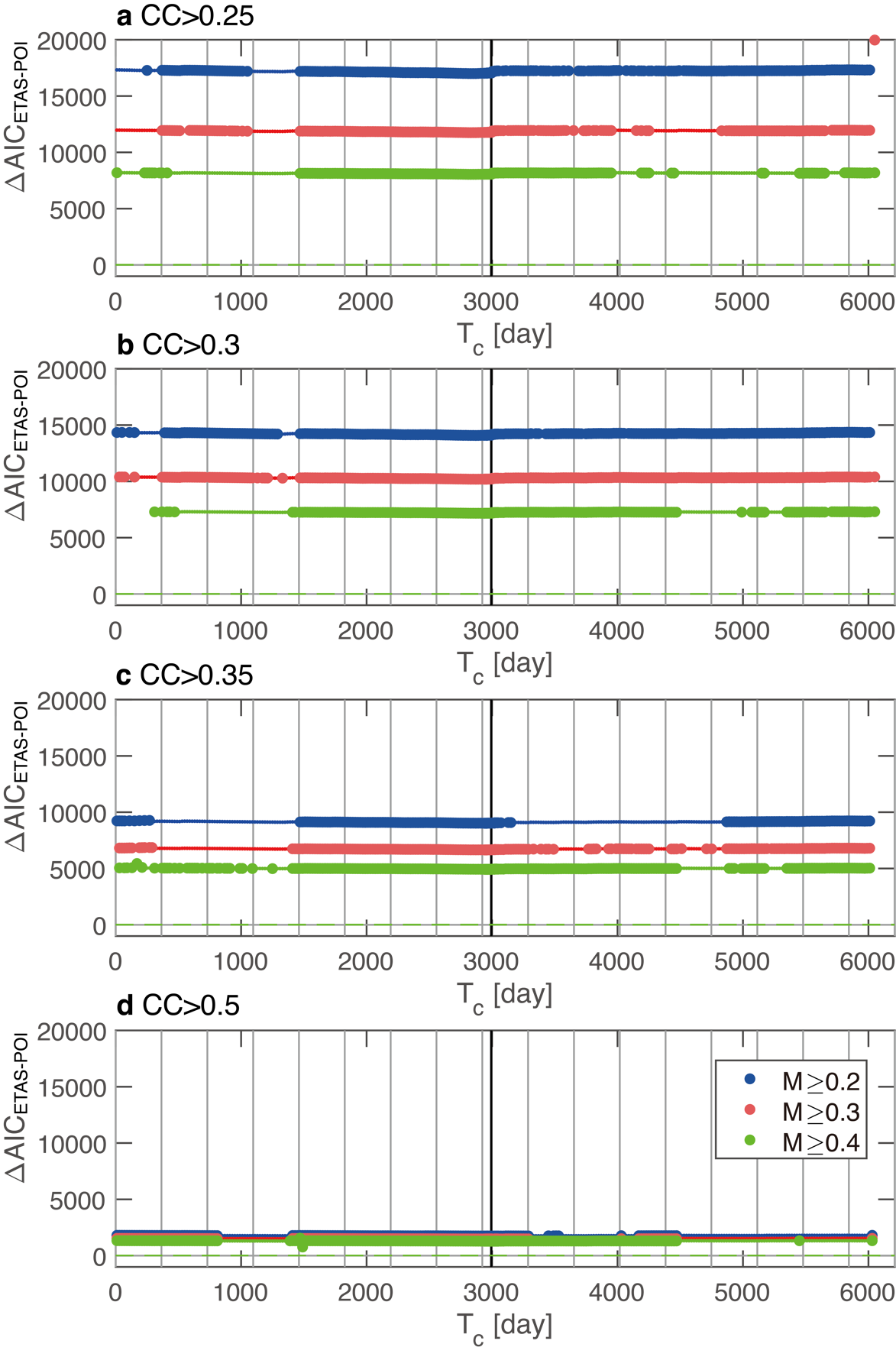


**Supplementary Fig. S6.** Comparison between the two-stage ETAS and Poisson models. The latter model is the same as the former, except for *K*_0_=0 (see the “ETAS model” section in Methods). This model assumes different parameter values for μ in subperiods before and after *T*_c_. Subtraction of AIC_2stage_ (AIC for the two-stage ETAS model) from AIC for the two-stage Poisson model gives ΔAIC_ETAS-POI_. **a**, ΔAIC_ETAS-POI_ (*CC*>0.25) as a function of *T*_c_ for different values of the minimum magnitudes: *M*_th_=0.2 (blue), 0.3 (red) and 0.4 (green). Small points show that the model-fitting analysis did not converge for either or both of the models when assuming the corresponding *T*_c_. **b**,**c**,**d**, Same as **a** for *CC*>0.3, 0.35, and 0.5, respectively. Also see the captions of Fig. 2 and Supplementary Fig. S5.


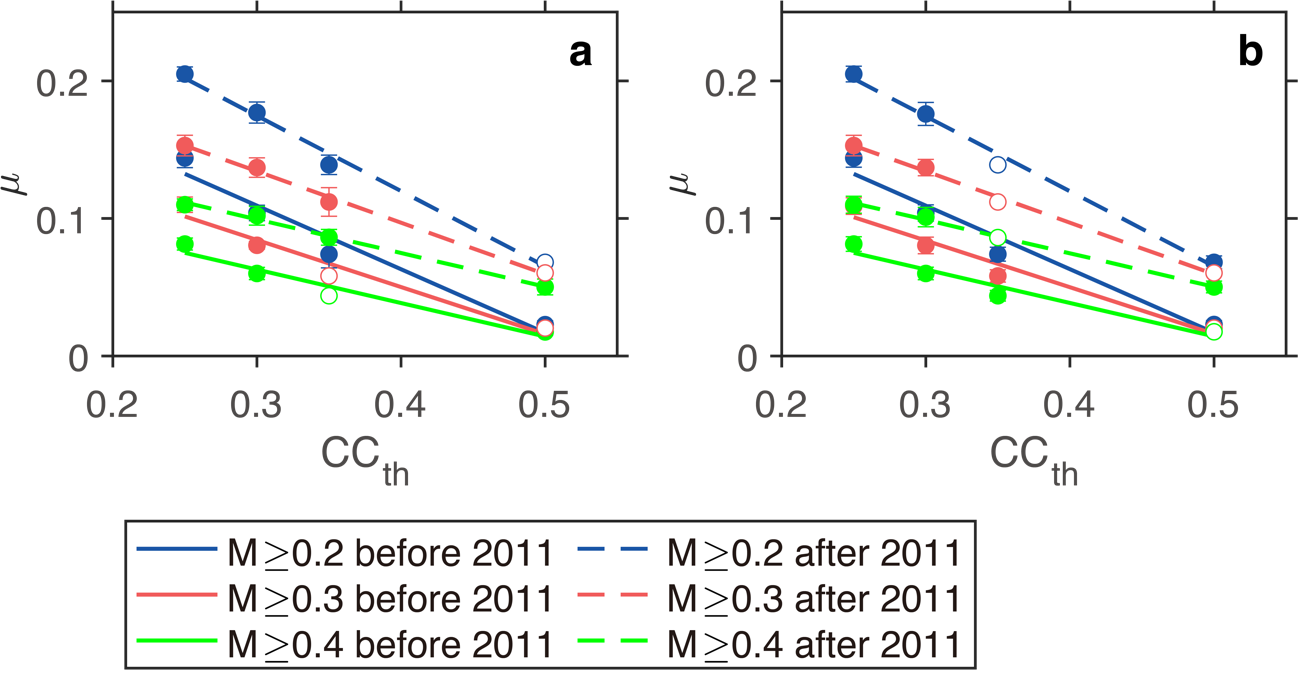


**Supplementary Fig. S7.** Sensitivity test of the μ-*CC*_th_ pattern. **a**, Same as Fig. 4**a** except that sets of parameters were prefixed as follows: (α, *K*_0_, *c*)=(0.5, 10^-5^, 0.0015). The slope (*g*) and intersection (*h*) of the least-square regression line and the square of the sample correlation coefficient (*R*^2^) are μ=*gCC*_th_+*h­* with (*g*, *h*, *R*^2^)=(-0.46, 0.25, 0.96) for *M*≥0.2 (blue solid line), (-0.34, 0.19, 0.96) for *M*≥0.3 (red solid line), and (-0.24, 0.14, 0.95) for *M*≥0.4 (green solid line) before 2011, and (-0.55, 0.34, 0.99) for *M*≥0.2 (blue dashed line), (-0.37, 0.25, 1.00) for *M*≥0.3 (red dashed line), and (-0.24, 0.17, 1.00) for *M*≥0.4 (green dashed line) after 2011. **b**, Same as **a** except that the sets of parameters were prefixed as follows: (*K*_0_, *c*, *p*)=(10^-5^, 0.0015, 2.8). μ=*gCC*_th_+*h­* with (*g*, *h*, *R*^2^)=(-0.46, 0.25, 0.96) for *M*≥0.2 (blue solid line), (-0.34, 0.19, 0.96) for *M*≥0.3 (red solid line), and (-0.24, 0.14, 0.95) for *M*≥0.4 (green solid line) before 2011, and (-0.55, 0.34, 0.99) for *M*≥0.2 (blue dashed line), (-0.37, 0.25, 1.00) for *M*≥0.3 (red dashed line), and (-0.24, 0.17, 1.00) for *M*≥0.4 (green dashed line) after 2011. See the “Time-dependent μ and *K*_0_” section in Methods, Supplementary Tables S2 and S3, and the caption of Fig. 3.


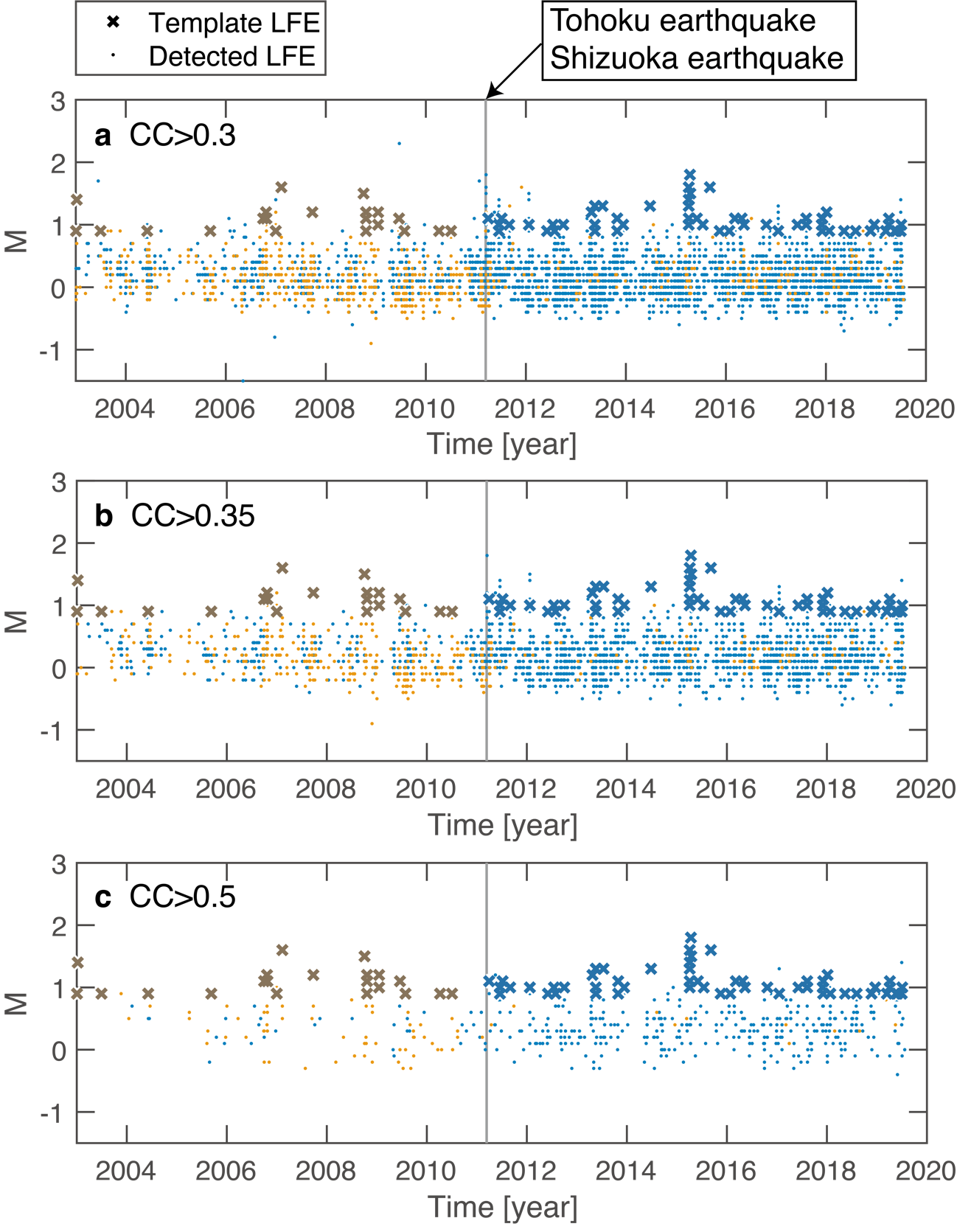


**Supplementary Fig. S8.** Same as the top panel of Fig. 5 for *CC*>0.3 in **a**, 0.35 in **b**, and 0.5 in **c**. Also see the caption of Fig. 5.


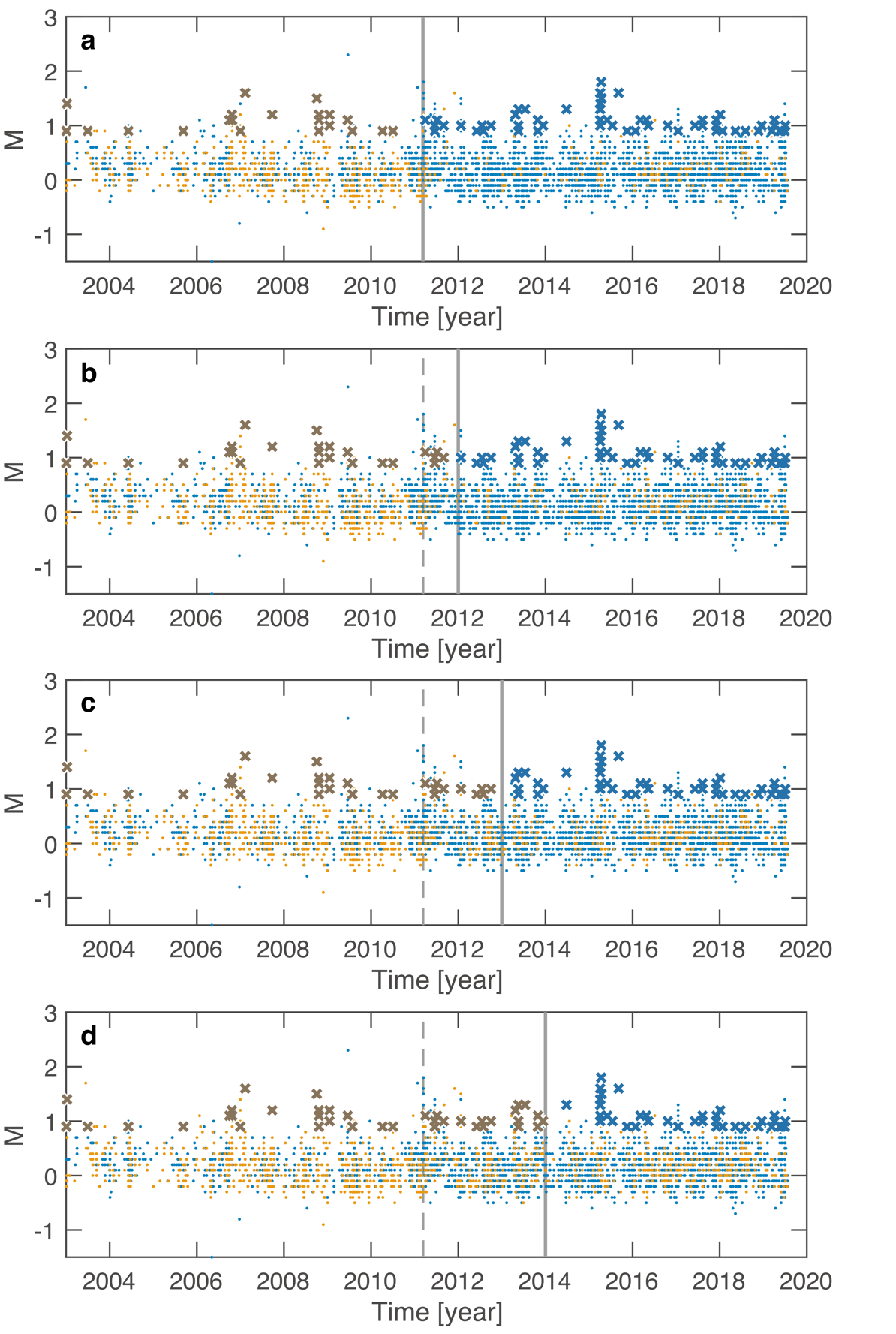


**Supplementary Fig. S9.** Same as Fig. 5 except for the time-windows, which were defined differently. **a**, Same graph as the top panel of Fig. 5. The same test as **a** was conducted for a time-limit (vertical solid line) before and after 2012 in **b**, 2013 in **c**, and 2014 in **d**. Vertical dashed line indicates the moments of Tohoku and Shizuoka earthquakes, which overlap with each other. In **a**, the majority of LFEs in the time-window after the time-limit appears to be colored in blue. Since the time-limit was set to 2014 in **d**, the majority of LFEs in the same time-window appears to be colored in both blue and orange. This indicates that templates (crosses) until 2014 from the moments of the Tohoku and Shizuoka earthquakes contributed to the detection of LFEs (dots) after 2014. These results support our statement that LFEs before/after the moments of the Tohoku and Shizuoka earthquakes were mostly detected by templates in the same time periods.
